# Fabricating Fibers of a Porous-Polystyrene Shell and Particle-Loaded Core

**DOI:** 10.3390/molecules24224142

**Published:** 2019-11-15

**Authors:** Dharneedar Ravichandran, Weiheng Xu, Rahul Franklin, Namrata Kanth, Sayli Jambhulkar, Sumedh Shukla, Kenan Song

**Affiliations:** 1 System Engineering, The Polytechnic School (TPS), Ira A. Fulton Schools of Engineering, Arizona State University, Mesa, AZ 85212, USA; dravich2@asu.edu (D.R.); weihengx@asu.edu (W.X.); sjambhul@asu.edu (S.J.); 2 Materials Science & Engineering, School for Engineering of Matter, Transport and Energy (SEMTE), Ira A. Fulton Schools of Engineering, Arizona State University, Tempe, AZ 85281, USA; rjfrank2@asu.edu (R.F.); nkanth@asu.edu (N.K.); 3 Manufacturing Engineering, The Polytechnic School (TPS), Ira A. Fulton Schools of Engineering, Arizona State University, Mesa, AZ 85212, USA; sshukl21@asu.edu; 4 The Polytechnic School (TPS) & School for Engineering of Matter, Transport, and Energy (SEMTE), Ira A. Fulton Schools of Engineering, Arizona State University, Mesa, AZ 85212, USA

**Keywords:** fibers, polymers, porous, composites, polystyrene, polyethylene glycol, spinning

## Abstract

Polystyrene (PS) polymers have broad applications in protective packaging for food shipping, containers, lids, bottles, trays, tumblers, disposable cutlery and the making of models. Currently, most PS products, such as foams, are not accepted for recycling due to a low density in the porous structure. This poses a challenge for logistics as well as creating a lack of incentive to invest in high-value products. This study, however, demonstrated the use of a dry-jet wet-spinning technique to manufacture continuous PS fibers enabled by an in-house designed and developed spinning apparatus. The manufactured fibers showed porosity in the shell and the capability to load particles in their core, a structure with high potential use in environmentally relevant applications such as water treatment or CO_2_ collections. A two-phase liquid-state microstructure was first achieved via a co-axial spinneret. Following coagulation procedures and heat treatment, phase-separation-based selective dissolution successfully generated the porous-shell/particle-core fibers. The pore size and density were controlled by the porogen (i.e., PEG) concentrations and examined using scanning electron microscopy (SEM). Fiber formation dynamics were studied via rheology tests and gelation measurements. The shell components were characterized by tensile tests, thermogravimetric analysis, and differential scanning calorimetry for mechanical durability and thermal stability analyses.

## 1. Introduction

Polymer fibers have traditionally been used in industries, such as textile [1], telecommunication [2], biomedical [3], construction [4], and electronics [5] industries, among many others. The characteristics of polymer fibers can be tailored by combining multiple dissimilar materials that have complementary properties. Recently, the study of bicomponent fibers has gained significant momentum. Bicomponent fibers involve combining two dissimilar materials to form a fiber and effectively using the features of both materials in related applications. The introduction of different phases in bicomponent fibers has also gained interest, not only for industrial uses but also to enhance their performance in tissue engineering [6], drug delivery [7], filtration [8], photocatalysis [9], supercapacitor electrodes [10], lithium-ion battery anodes [11], three-dimensional (3D) printing [12], and artificial turf [13]. Based on the nature of the polymer and additional fillers, the properties of bicomponent fibers such as mechanical stiffness/strength [14], electrical/thermal conductivity [15,16], optical reflectivity/absorbance [17], self-healing/-cleaning [18], and other stimuli-response behaviors can be altered. These properties can be used to a) isolate an unstable component thereby reducing the chance of decomposition under a reactive or extreme environment (e.g., water treatment); b) release core materials with controlled rates to a particular receptor (e.g., drug delivery); and c) increase the mechanical durability or integrity for functional behaviors (e.g., multiphase composites), as well as, (d) various other applications for sensing or actuating (e.g., smart sensors and soft robotics).

Bicomponent fibers can be classified into three categories: core/shell or core/sheath (C/S), side-by-side (S/S), and islands-in-the-sea (I/S) [19]. Among these, the permeable shell of C/S microstructured fibers has the most widespread applications in catalysis, drug delivery, water treatment, carbon dioxide (CO_2_) absorption and storage, scaffolds, filtration, and sensor fabrication due to a high surface area, which maximizes the surface contact of the core materials with the surrounding environment [20]. These fibers, in the form of one polymer surrounded by another polymer or particles encapsulated in a matrix, can be further modified by introducing pores on the surface or at interfaces. Many reported methods can generate pores in general polymeric structures, namely colloidal templating or direct templating [21], block copolymer self-assembly [22], direct synthesis [23], high internal phase emulsion polymerization [24], interfacial polymerization [25], breath figures [26], vapor/nonsolvent/thermally induced phase separation [27,28], and selective dissolution [29], among many others. Very recently, Lackner, etc. used sorbent-filled disks to bind up CO_2_ in the air [30]; however, the efficiency is limited by the contact between absorbent PS and the air in disks and the media containing absorbent with porous microstructures is predicted to significantly improve the CO_2_ capture. For the current study, scalability and the unique fiber form were considered; thus, porogen incorporation and phase inversion based on the facile selective dissolution of one phase from the other were used for pore formation. The pore size and distribution was controlled via the porogen concentration and etching procedures.

Previous research reporting fabrication of porous and nonporous C/S fibers [31,32,33,34] have mostly used an electrospinning method where the electrical conductivity of the solution and the dielectric constant of the solvent are two of the critical determining factors. However, a lack of electrical conductivity in polymer melts or highly concentrated ceramic suspensions leads to inefficient fiber formation and morphological defects [35,36,37]. The challenges of scalability, collectivity, and post-treatment capability on single filaments also limit the application of electrospinning to the biomedical, filtering and separation, and energy generation fields. In contrast, large-scale fiber spinning technique such as a dry-jet wet-spinning method is more flexible in different processing conditions, thus was chosen for this study. The processing conditions for dry-jet wet-spinning [38,39,40,41,42] include:

(a) design of the spinneret (e.g., fiber morphology control),
(b) choice of different materials (e.g., two or more phases of fibers, micro-fillers, and nanoparticles),
(c) coagulation processes (e.g., bath temperatures, compositions, concentrations, and soaking time for fiber formations and producing even surface coatings),
(d) filament windability (e.g., manufacturability and polymer morphology controls),
(e) post-treatment steps (e.g., annealing, quenching, stress-applied healing, etc.).

From among seven different resin materials, PS with a plastic recycling number or resin identification code of ‘6’ [43], is one of the least recycled polymers. Conventional methods of chemical disposal of PS include landfilling, incineration, and recycling. Landfilling is not desirable due to its harmful environmental consequences [44] and similarly, burning causes air pollution [45]. Recycling is more environmentally friendly but is often costly due to the extensive involvement of plastic separation and the low-value product market [46]. This study explored the use of PS polymers, including the spinning of PS waste foams, in an environmentally-related material system. Specifically, this paper reports a simple fabrication technique to produce fibers with core/shell morphology with controlled porous structures and particle loading capabilities via a uniquely in-house designed dry-jet wet-spinning process. PS pellets (PS_p_) were used as a matrix, micron-sized PS spheres (PS_s_) were used as loaded particles, while polyethylene glycol (PEG) served as both a porogen to create pores and a dispersing matrix to stabilize filled particles. The effect of rheology on fiber formation dynamics, the influence of porogen on controlling pore sizes and distribution of the shell, and the uses of different solvents on particle loading efficiency were studied. Specifically, this paper reports a simple fabrication technique to produce fibers with core/shell morphology with controlled porous structures and particle loading capabilities via a uniquely designed dry-jet wet-spinning process. This study aims to recycle low-value waste PS plastics for environmental applications, which can potentially be used for wastewater treatment or CO_2_ management.

## 2. Results and Discussions

### 2.1. Fabrication Design

The aim of this study was to fabricate porous-shell/hollow-core microstructures in fibers with particle loading capability. Therefore, careful consideration was given to the selection of two different polymers (polymer matrix and pore-forming porogen), which could

(a) allow the blended polymer mixtures to coagulate in the non-solvent to form fibers so that during the solvent/non-solvent exchanging process, the blended polymer solutions can transform from solution-state liquid to gel-state fiber precursors,
(b) selectively dissolve the porogen in a solvent to create pores,
(c) evenly distribute particles in the hollow cores,
(d) simultaneously avoid losing particles from the constructed porous surfaces or hollow centers, (e.g., particle sizes should be smaller than the pore and be constrained by the core without flowing out).

**Figure 1:**
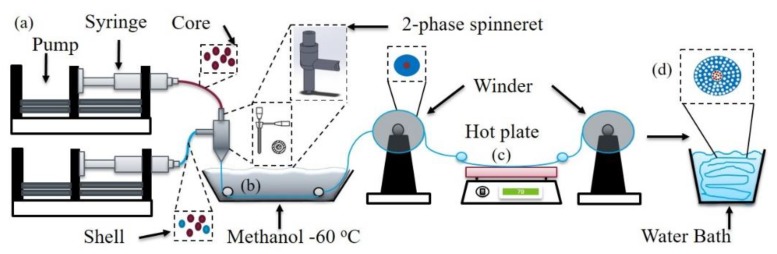
(**a**) Dry-jet wet-spinning of solution injection via an in-house designed two-phase spinneret, (**b**) coagulation, (**c**) heat treatment on the hot plate, and (**d**) porogen agent dissolution and pore generation.

PS [47] and PEG [48] were thus used as material examples, as both can be dissolved in a common solvent (e.g., xylene and dimethylformamide) without phase separations. Because PEG can be dissolved in water, it can be selectively etched in aqueous soaking for the shell pore formations or to allow the core to contain homogeneously distributed particles. Figure 1 shows the dry-jet wet-spinning process that formed the bicomponent shell/core structures, with a unique, in-house designed and 3D printed spinneret (Figure 1a). The 3D printing allowed the rapid prototyping of the spinneret with appropriate size and complex architectures, and, durable plastics resistant to chemicals and high temperatures. The fibers went through coagulation to form gel-fibers (Figure 1b), had heat treatment to eliminate solvent and non-solvent (Figure 1c), and eventually were soaked in water to create pores in the shell and retain particles in the core (Figure 1d). Two kinds of PS_p_, namely low molecular-weight PS (i.e., PS_p-LM_ with an average molecular-weight (Mw) of 192,000 g/mol) and high molecular-weight PS (i.e., PS_p-HM_, Mw of 350,000 g/mol), were used. The two PS polymers were chosen to examine the influence of molecular-weight on fiber formation feasibility and mechanical durability. The fabricated fibers of different compositions and microstructures and their nomenclature are detailed in the experimental Section 3.2.

### 2.2. Influence of Rheology on Fiber Formations

**Figure 2:**
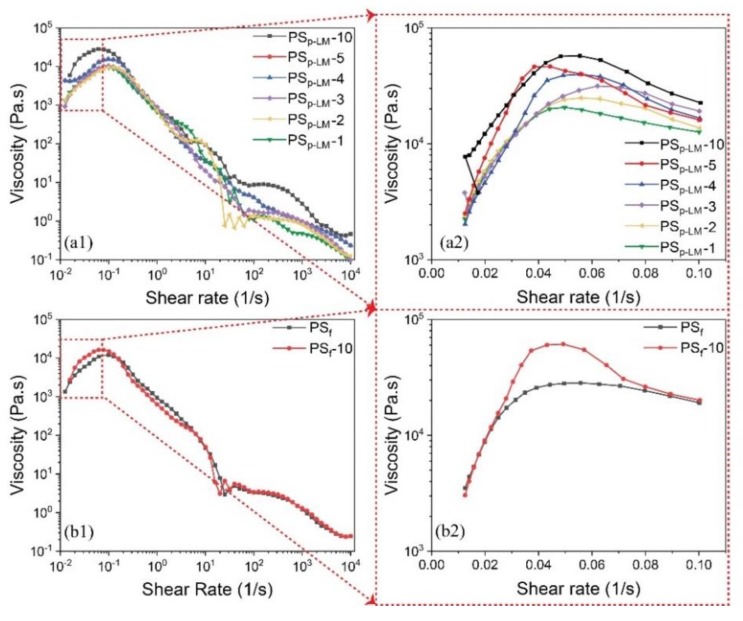
Polyethylene glycol (PEG)/polystyrene (PS) of (**a1**) 1, 2, 3, 4, 5, and 10 wt. % using low-molecular-weight PS pellets (PSp-LM) and (**a2**) zoom-in regions at lower shear rates. (**b1**) 10 wt. % using PS-based packaging foams (PSf) and (**b2**) zoom-in regions at lower shear rates.

A rheology test was conducted to observe the solution viscosity affecting fiber formation and morphology [49,50]. Viscosity refers to the proportionality constant between shear stress and shear strain. Figure 2(a1,a2) show the viscosity change at room temperature (25 °C) as a function of shear rate (10^−2^ to 10^4^ 1/s) for 35 wt. % PS_p-LM_/xylene solution with a varying concentration of PEG (e.g., 1, 2, 3, 4, 5, and 10 wt. % with respect to PS_p_). PS_p-LM_ in xylene with lower concentrations was also prepared but failed to form fibers, proving that the 35 wt. % concentration was within the semi-concentrated to concentrated polymer solution range, allowing the polymer chains to entangle with each other for fiber formations. The change in viscosity had a similarly increasing trend for all concentrations at a lower shear rate (0.01–0.05 1/s), followed by a quasi-plateau region until 0.10 1/s. As the shear rate increased, shear-thinning was consistently observed until a shear rate of 10^4^ 1/s was obtained. The rheology curves suggested non-Newtonian behaviors for the polymer solutions used for spinning. According to the Flory-Huggins equation, concentrations of the polymers in the solution will significantly affect the viscosity [51]. Spencer and Williams also verified the viscosity behavior of concentrated PS solution in different solutions (e.g., with a high PS_p-LM_ concentration of 35 wt. %) and concluded that Flory’s relationship is valid [52,53]. At a higher shear rate (10^2^–10^3^ 1/s), a sudden increase in the viscosity in the PS_p-LM_-10 (i.e., 10 wt. % PS_p-LM_ in xylene) solution was noted. This usually occurs due to molecular structure rearrangement from the applied shear, and it is referred to as flow-induced shear-thickening [54]. This phenomenon was observed in all sample solutions at different shear rates, being most significant at higher concentrations of PEG. The addition of PEG slightly increased viscosity (Figure 2(a1)), especially at a lower shear rate (Figure 2(a2)). Solution spinning is usually done in the shear-thinning region between 1–10^3^ 1/s, and the observed viscosity demonstrated the feasibility of using the prepared solution batches for fiber spinning (Figure 2(a1)) [55]. It is noteworthy a key goal of our fibers was the application for environmental sustainability, such as recycling PS foams from solid waste (PS_f_); thus, PS_f_ that was directly collected from waste food packaging materials and examined to study their spinnability. The PS_f_ materials were used as obtained to prepare spinning dopes. Similar observations of shear-thinning behaviors were found for 35 wt. % PS_f_ in xylene both with and without 10 wt. % of PEG (PS_f_-10), as seen in Figure 2(b1,b2), demonstrating a similar feasibility of using PS_f_ for fiber spinning to commercialized PS_p_.

### 2.3. Influences of PEG Concentration on PS Porous Microstructures

**Figure 3:**
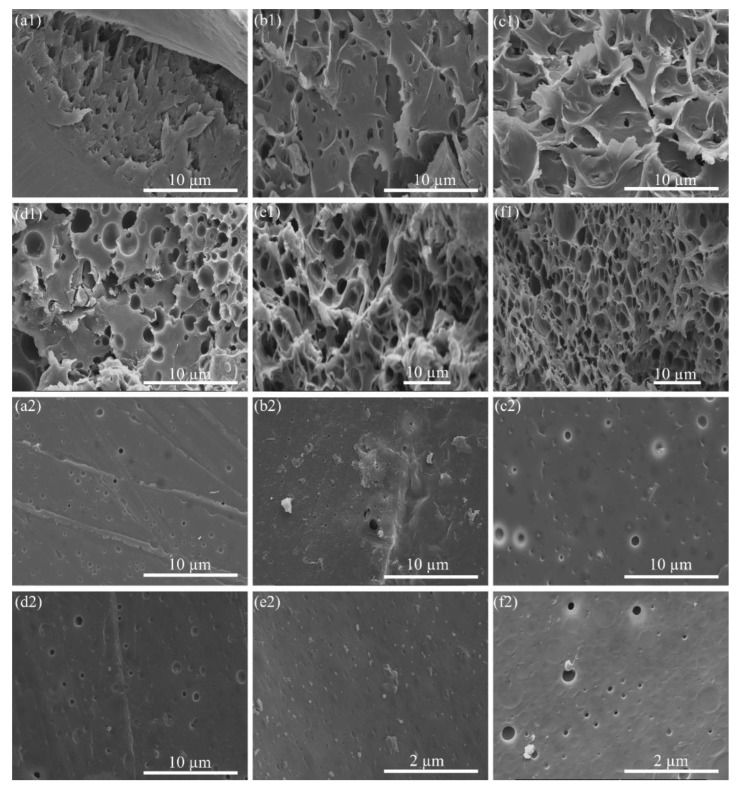
SEM images showing 35 wt. % PS_p-LM_/PEG of (**a**) 1 wt. %, (**b**) 2 wt. %, (**c**) 3 wt. %, (**d**) 4 wt. %, (**e**) 5 wt. %, and (**f**) 10 wt. % immersed in the water against the flow to dissolve PEG for pore generation. (**a1**–**f1**) Cross-section areas and (**a2**–**f2**) surface areas. Figures S1 and S2 show an enlarged view of (**e2**,**f2**).

The PS_p_/PEG mixtures in xylene were injected into the coagulant to form fibers, after which they were placed in water flow for 24 h at room temperature to etch the PEG phase. After dissolving different concentrations of PEG, SEM images of PS_p-LM_ porous structures illustrated the pore sizes and distributions on both cross-sections and surface areas (Figure 3 and Figures S1–S3). On the cross-section area, the PEG concentration of PS_p-LM_-10 clearly showed much larger pores as compared to the lower PEG concentrations (Figure 3(f1), Figure S2). The porous directions were also associated with nanofibrils aligned along the fiber axis, as a result of the uniformity of pores and separation among individual PS fibrils. The diameter of the pores generated varies from tens of nanometers to a few micrometers. With the increased concentration of PEG from 1 wt. % to 10 wt. %, the pores became more homogeneous across the cross-section. While the fiber surfaces (the out layer of fibers) had similar trends, the surface pore distribution exhibited much less density compared to the cross-section at the same PEG concentration with the limited soaking time. The difference between Figure 3 and Figure S3 was the soaking method, with the former fiber soaking in the water against the flow while the latter in the static water system, demonstrating a more efficient pore generation mechanism with water flowing, thereby bringing dissolved PEG along.

**Figure 4:**
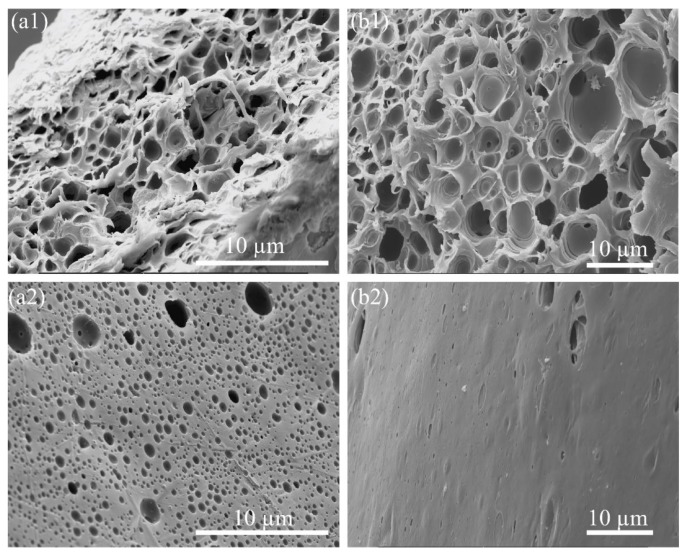
SEM images showing (**a**) 10 wt. % PEG/35 wt. % PS_p-HM_ and (**b**) 10 wt. % PEG/35 wt. % PS_f_ solutions were injected as fibers and soaked in water for 24 h. (**a1**,**b1**) Cross-section areas, and (**a2**,**b2**) surface areas.

Similar to the process of fabricating PS_p-LM_, the solution preparation, fiber fabrication processes, and the testing parameter, the pore formation in the cross-section and the surface area were the same for PS_p-HM_ and PS_f_ respectively. Due to the high porosity and the challenges in fabricating collectable and durable fibers, a 10 wt. % concentration of PEG was consistently used in Figure 4. SEM images in Figure 4 demonstrate that PS_p-HM_ had a more homogeneous distribution of pores in both cross-section and surface area, with pore size varying from a few nanometers to micrometers. In contrast, PS_f_ had features similar to the PS_p-LM_/PEG mixture. While PS_p-HM_ showed better control of pore size and distribution as well as better mechanical and thermal properties, PS_p-LM_ was the low critical to fabricate mechanically durable fibers. In other words, PS_p-HM_ will be produced as fibers without technical issues if the PS_p-LM_ can be successfully spun and collected. Thus, PS_p-LM_ was selected to study the characterizations of thermal stability and particle loading capabilities.

### 2.4. Thermal Transitions of Fabricated Materials

Differential scanning calorimetry (DSC) and Thermogravimetric analysis (TGA) were used to characterize the thermal transitions of as-obtained raw materials and processed fibers, as well as to identify their compositions. As shown in Table 1 (curves in Figures S4 and S5), PS exhibited an amorphous structure, whereas PEG demonstrated a crystalline structure (i.e., DSC curves), with the latter being less stable than the former (e.g., TGA transitions of PEG were much smaller than that of PS). The lack of crystallinity caused the fabricated fibers to be brittle and confirmed the fiber formation mechanism of polymer chain entanglements. PS_p-LM_-10 showed limited crystallinity due to the majority of PS in the blends and its influence on the PEG crystallization process. The soaking of these blend fibers removed some PEG but did not eliminate the entire PEG content, as the enthalpy of crystallization dropped from 10.32 J/g in PS_p-LM_-10 fibers to 7.13 J/g after soaking the porous PS_p-LM_-10 fibers in water. Compared to the pure PS_p-LM_, the PS_p-LM_-10 with 10% PEG composition-based fibers underwent a two-step degradation process during TGA, with the first degradation taking place between 130 °C–145 °C, suggesting presence of solvent residue from coagulation even after heat treatment (the boiling point of xylene is 138.4 °C) (T_i1_ in Table 1).

### 2.5. Influence of the Solution on Particle Loading

**Figure 5:**
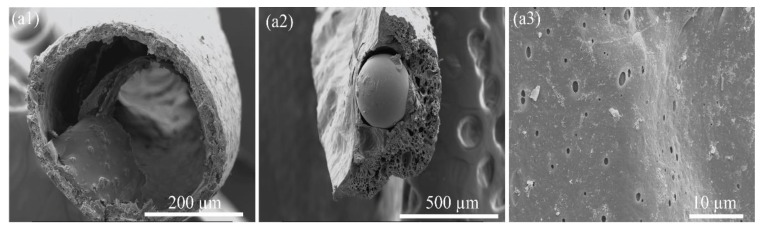
(**a1**) Core/shell structure from PSp-LM/xylene/PEG that lost PSs, (**a2**) core/shell structure using PSp-LM/DMF/PEG that retained PSs and (**a3**) surface morphology.

Notice that in environmentally relevant applications such as water treatment [56,57] or CO_2_ capture [58], micro- or nanoparticles have been generally included as catalyst or absorbent. PS_s_ were used as an example of particles loaded in the PS_p-LM_ fibers. As solid particles, these PS_s_ need to be dispersed in a liquid or gel media to become loaded in the fiber core. The center of the developed spinneret (Figure 1) was therefore injected with the PS_s_ dispersed in PEG/water solutions. Instead of xylene, water was used to dissolve PS_s_ to retain the spherical shapes; also, the solution was injected at room temperature to avoid the diffusion to the core area of xylene from the shell channels (Figure 1) and a resulting dissolution of PS_s_. To evenly distribute the PS_s_ in the core, a highly viscous PEG/water solution was prepared with an 80 wt. % PEG concentration. The injection of the exterior layer (e.g., PS_p-LM_/xylene/PEG) and the interior layer (e.g., PEG/water/PS_s_) into the spinneret and coagulation of the injected fibers produced the core-shell structure shown in Figure 5(a1). However, due to the long coagulation time to form continuous fibers, the PS_s_ were found to be absent from the porous fibers.

During the core/shell spinning process, the coagulation speed of the shell fiber is critical to prevent any loss of core particles, as the particles contained in the core with PEG solutions do not form a fiber. During coagulation, xylene was not efficiently exchanged with the coagulant methanol, as confirmed by the TGA data after 24 h of coagulation. Therefore, the solvent dimethylformamide (DMF), was used for faster coagulation rates to examine its influence on fiber formations and particle retention, as it has superior miscibility with water and many other organic solvents. The PS_p-LM_/DMF/PEG solution of the same composition (i.e., PS_p-LM_ 35 wt. % in DMF with PEG 10 wt. % with respect to PS_p-LM_) was able to coagulate in methanol at room temperature much faster, preventing the particles from washing away during the coagulation and pore generation processes (Figure 5(a2,a3)). Use of DMF was also found to influence the as-spun fiber dimensions, as the same spinning conditions—including the injection rates—generated the core sizes of ~400 µm in diameter using xylene, while the use of DMF produced fiber cores of ~300 μm that confined the PS_s_ in the core areas.

The use of DMF instead of xylene also improved mechanical durability due to shorter coagulation periods. Fibers fabricated using PS_p-LM_/xylene/PEG were brittle and weak; while those fabricated using PS_p-LM_/DMF/PEG solution showed consistent elastic modulus and tensile strength, as reported in Table S1. Please note that the fibers were highly porous and the real cross-sections bearing the applied load were much smaller, such that the intrinsic stiffness and strength were much higher than the tested values considering the porosity and pore interconnectivity. The mixing of flexible, crystalline polymers and reinforced particles can also enhance the mechanical properties.

## 3. Materials and Methods

### 3.1. Materials

Low molecular-weight PS pellets (PS_p-LM_, Mw of 192,000 g/mol, melt flow index 6.0–9.0 g/min, SKU# 430102, CAS #9003-53-6), high molecular-weight PS pellets (PS_p-HM,_ Mw of 350,000 g/mol, average number-based average molecular weight (Mn) of 170,000, melt index 2.0–4.0 g/min, SKU# 441147, CAS #9003-53-6) and PEG (Mw, Mn of 10,000, SKU# 309028, CAS #25322-68-3) were purchased from Sigma Aldrich (St. Louis, Missouri, USA). Xylene (>98.5%, boiling point (BP) ~137–140 °C, vapor pressure (Vp) at 37.7 °C ~2.4 kPa, CAS #1330-20-7) were purchased from Sigma Aldrich (St. Louis, Missouri, USA). Methanol (>95%, BP 64.7 °C, Vp at 20 °C ~12.8 kPa, CAS #67-56-1) was purchased from ThermoFisher Scientific (Waltham, Massachusetts, USA). Dimethylformamide (>95%, BP 153 °C, Vp at 20 °C ~0.49 kPa, CAS #68-12-2) and PS spheres (PS_s_, Bp 100 °C, Vp at 20 °C ~0.93 kPa, CAS #069011-18-3) were purchased from Fisher Chemicals (Hampton, New Hampshire, USA) and Epicor Incorporated (Linden, New Jersey, USA) respectively. All the polymers and solvents were used as received. PS_f_ were obtained from waste plastics and used without treatment.

### 3.2. Experimental Fabrications

A two-phase co-axial spinneret was designed and manufactured using a Concept Laser Mlab metal 3D printer (Lichtenfels, Germany) with cobalt-nickel resin (Figure 1). To prepare porous fiber shells, 35 wt. % of PS_p_ was dissolved in xylene at 120 °C using a mechanical stirrer. Next, 1, 2, 3, 4, 5, and 10 wt. % of PEG was dissolved in the PS_p_/xylene solution using the mechanical stirrer at the same temperature (120 °C). To form fibers with hollow cores, 80 wt. % of PEG was dissolved in water at 110 °C using a magnetic stirrer. The PS_p_/xylene/PEG and the PEG/water solutions were injected separately to the core and shell, as shown in Figure 1a. To form the fiber core with loading capabilities, particles were added to the core materials of PEG/water 80 wt. % solutions for stabilized suspensions. For example, after completely dissolving PEG in water, the mixture was cooled down to room temperature (25 °C), and 30 wt. % of PS_s_ was added to the PEG/water solution and uniformly dispersed using a magnetic stirrer. Water was used in the core solution as a dispersion medium to DMF-based solution was prepared in the same method with the same concentration to understand the solution behavior during coagulation. The composition and the nomenclature are listed in Table 2.

Figure 1 shows the schematic of the dry-jet wet-spinning setup employed for the core/shell fiber fabrication. After the shell and core solutions were obtained, they were placed separately in two syringes fed into spinneret channels by syringe pumps. The shell solution and the core solution were connected to the exterior and interior outlets of the spinneret, respectively. The solutions were pumped at different rates to accommodate the diameter changes in the outer and inner outlets and the wall thicknesses of the spinneret. The composite first went through a coagulation bath of methanol maintained at −60 °C using dry ice. A winder setup was used to collect the incoming fibers from the coagulation bath. The fibers were then drawn using a two-winder setup rotating at a 1:1 speed-ratio at 100 °C to remove the remaining solvents, increase the mechanical strength, and attain high orientation during their solidification. The fibers were then kept in a water bath to remove PEG from the shell and/or the core in order to form hollow cores and porous regions.

### 3.3. Characterizations

The morphology, pore formation, and pore distribution of fibers were studied using a scanning electron microscope (SEM) (XL30 ESEM-FEG) (Amsterdam, Netherlands). An Au/Pt nanoparticle layer of thickness 15 nm was sputter-coated on fiber surfaces to improve conductivity. TGA (TGA550, TA Instruments) (New Castle, Delaware, USA) was conducted in a nitrogen environment at a heating rate of 10 °C/min. Nitrogen was purged at 40 mL/min for 10 min to remove the air from the testing chamber before tests. DSC (DSC250, TA Instruments) (New Castle, Delaware, USA) was conducted in a nitrogen environment with a purging rate of 50 mL/min. A heating rate of 5 °C/min was used for the processes of heating from 50 °C to 200 °C and cooling from 200 °C to −50 °C. A rheology test (Discovery Hybrid Rheometer HR-2, TA Instruments) (New Castle, Delaware, USA) was conducted on the PS_p-LM_/xylene/PEG shell solutions to study the effect of temperature and time on the spinning dynamics by using a 25 mm steel parallel plate with environmental test chamber (ETC) at a shear rate of 10^−2^ to 10^4^ 1/s and a 100 µm gap at room temperature. Tensile test (Discovery Hybrid Rheometer HR-2, TA Instruments) (New Castle, Delaware, USA) was performed on the shell fiber made of PS_p-LM_/DMF/PEG to study the mechanical properties of the fiber using a rectangular tension fixture with ETC at a constant linear rate of 10 µm.

## 4. Conclusions

In this study, porous PS shells with loaded particles were successfully fabricated using a dry-jet wet-spinning technique. The weight percentage of PEG in the shell solution proved to be an important parameter that influenced both pore size and distribution. The pores were induced by phase separation of PEG via the selective dissolution of the fiber in water. The concentration of PEG also affected the mechanical strength of the fibers and the viscosity of the shell solution. Viscosity had a crucial impact on the solution flow during the spinning process and the packing capability of the core. TGA and DSC analyses explained the composition and crystallization character of individual fibers. This study fundamentally demonstrated the efficiency of particle loading capability for C/S fibers using dry-jet wet-spinning technique and explained the feasibility of fiber spinning using recycled PS waste.

## Figures and Tables

**Table 1:** Thermal transition temperatures for as-obtained raw materials and fabricated and post-processed fibers using DSC and TGA.

Samples	DSC			TGA			
	Tg (°C)	ΔH (J/g)	Tm (°C)	Main Peak Temperature of the Curve Derivative (°C)	T_i1_ (°C)	T_i2_ (°C)	T_f_ (°C)
**As-obtained raw materials**							
PS_p-LM_	102.07	-	-	410.11	-	287.29	430.61
PEG Flakes	-	179.49	63.94	366.35	-	218.60	384.56
**Fabricated and post-processed fibers**							
PS_p-LM_ fiber	101.62	-	-	337.06	132.49	287.86	370.24
PS_p-LM_-10 fiber	102.06	10.32	61.48	375.17	145.46	295.42	404.11
Porous PS_p-LM_-10 fiber	102.21	7.13	61.27	360.05	142.43	293.29	397.41

Note: Tg, glass transition temperature; ΔH, enthalpy of crystallization; Tm, melting temperature; T_i1_, first degradation temperature; T_i2_, second degradation temperature; T_f_, final degradation temperature.

**Table 2:** Sample nomenclature.

Sample	PEG/P_S_ wt. % in Shells	PEG/Water wt. % in Core	Particle/PEG wt. % in Core	Preparations
PS_p-LM_-1	1	0	0	(i) A 35 wt. % PS_p_ concentration in xylene was prepared as a solution; (ii) 1, 2, 3, 4, 5, and 10 wt. % PEG as a ratio to PS_p_/xylene solution was added to the solution as spinning batches; (iii) The spinning solutions were gel-spun as fibers and heat-treated; (iv) The solid fibers were soaked in the water to get dissolved PEG to form pores. The porous fibers were dried before further characterizations.
PS_p-LM_-2	2			
PS_p-LM_-3	3			
PS_p-LM_-4	4			
PS_p-LM_-5	5			
PS_p-LM_-10	10			
PS_p-LM_-10/PEG core	10	80	0	(v) Shell solutions as mentioned above; core solutions were 80 wt. % PEG/water.
PS_p-LM_-10/PS_s_	10	80	30	(vi) Shell solutions as mentioned above; 30 wt. % PS_s_/PEG was prepared for the core solutions.
PS_p-HM_-10	10	0	0	(vii) A 35 wt. % PS_p_ concentration in xylene with 10 wt. % PEG was prepared as a solution and followed step iii and iv.
PS_f_-10	10	0	0	(viii) 35 wt. % of PS_f_ concentration in xylene with 10 wt. % PEG was prepared as a solution and followed step iii and iv.

## Supplementary Materials

The following are available online, Figure S1, enlarged view of Figure 3(e2) for a better view of the pores on the surface area; Figure S2, enlarged view of Figure 3(f2) for a better view of the pores on the surface area; Figure S3, Polyethylene glycol(PEG)/polystyrene (PS_LM_) of (a) 1 wt. %, (b) 2 wt. %, (c) 3 wt. %, (d) 4 wt. %, (e)5 wt. %, and, (f) 10 wt. % PEG in PS_LM_ immersed in water flow to dissolve PEG for pore generation. a1-f1 demonstrate cross-section areas, and a2–f2 demonstrate surface areas; Figure S4, DSC curves; Figure S5, TGA curves; Table S1, mechanical properties of obtained and fabricated fibers at varying PEG concentrations.
