# Supplementary material for: Fabricating Fibers of a Porous-Polystyrene Shell and Particle-Loaded Core
Source: Molecules. 2019 Nov 15;24(22):4142. doi: 10.3390/molecules24224142 (PMC6891604; doi:10.3390/molecules24224142)
Supplement: Supplementary file 1 [file molecules-24-04142-s001.pdf]

Fabricating Fibers of a Porous-Polystyrene Shell and Particle-Loaded Core

Dharneedar Ravichandran[a], Weiheng Xu[a], Rahul Franklin[b], Namrata Kanth[b], Sayli Jambhulkar[a], Sumedh Shukla[c], Kenan Song[d, *]

a System Engineering, The Polytechnic School (TPS), Ira A. Fulton Schools of Engineering, Arizona State University, Mesa, AZ 85212, USA

b Materials Science & Engineering, School for Engineering of Matter, Transport and Energy (SEMTE), Ira A. Fulton Schools of Engineering, Arizona State University, Tempe. AZ 85281, USA

c Manufacturing Engineering, The Polytechnic School (TPS), Ira A. Fulton Schools of Engineering, Arizona State University, Mesa, AZ 85212, USA

d,* Assistant Professor, The Polytechnic School (TPS) & School for Engineering of Matter, Transport, and Energy (SEMTE), Ira A. Fulton Schools of Engineering, Arizona State University, Mesa, AZ 85212, USA; Email: kenan.song@asu.edu

Supporting Information

Table of Contents

Figure S1. Enlarged view of Figure 3(e2) for better a view of the pores on the surface area.

Figure S2. Enlarged view of Figure 3(f2) for better a view of the pores on the surface area.

Figure S3. Polyethylene glycol(PEG)/polystyrene (PS_{LM}) of (a) 1 wt%, (b) 2 wt%, (c) 3 wt%, (d) 4 wt%, (e)5 wt%, and, (f) 10 wt% PEG in PS_{LM} immersed in water flow to dissolve PEG for pore generation. a1-f1 demonstrate cross-section areas, and a2-f2 demonstrate surface areas

Figure S4. DSC curves

Figure S5. TGA curves

Table S1. Mechanical properties for obtained and fabricated fibers at varying PEG concentration

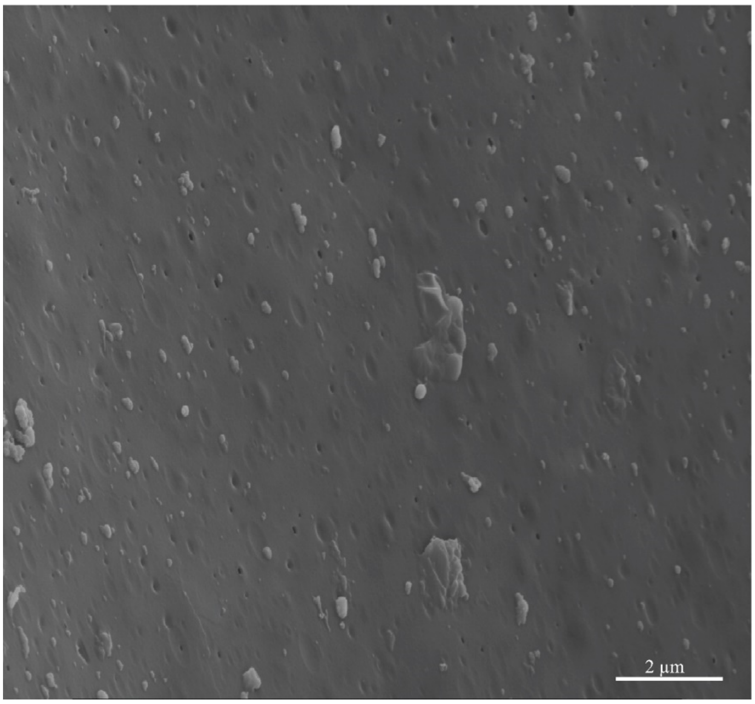

Figure S1. Enlarged view of Figure 3(e2) 35 wt% PS$_{p-LM}$ and 5 wt% PEG in xylene for better a view of the pores on the surface area.

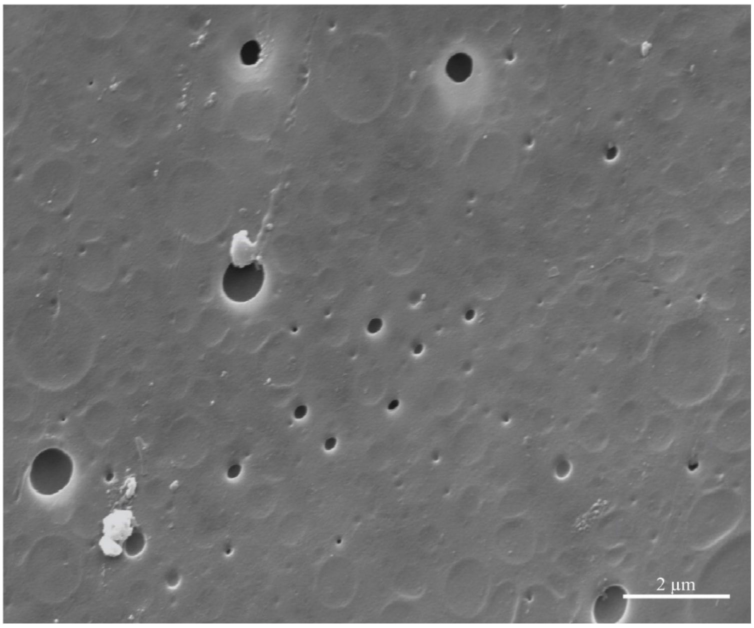

Figure S2. Enlarged view of Figure 3(f2) 35 wt% PS$_{p-LM}$ and 10 wt% PEG in xylene for better a view of the pores on the surface area.

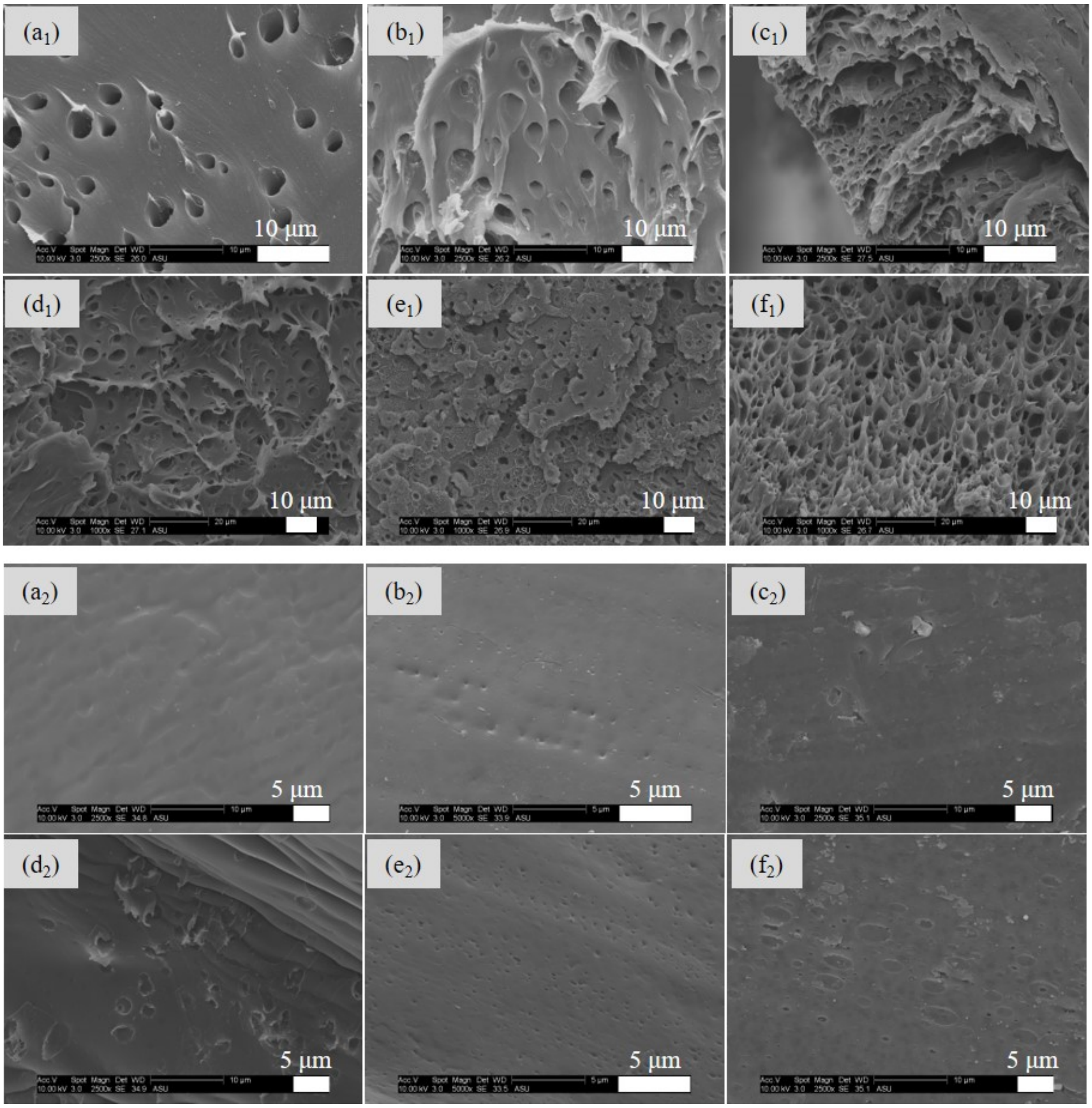

Figure S3. Polyethylene glycol(PEG)/polystyrene (PSLM) of (a) 1 wt%, (b) 2 wt%, (c) 3 wt%, (d) 4 wt%, (e)5 wt%, and, (f) 10 wt% PEG in PSLM immersed in water flow to dissolve PEG for pore generation. a1-f1 demonstrate cross-section areas, and a2-f2 demonstrate surface areas

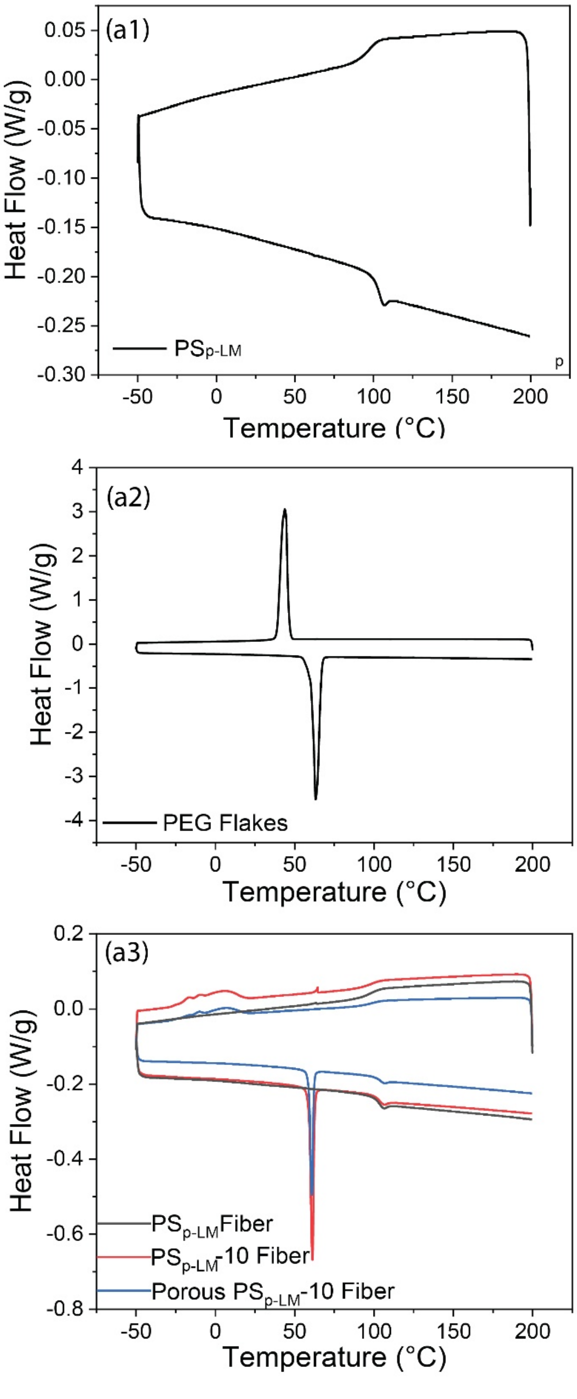

Figure S4. DSC curves for (a1) as- purchased $PS_{p\text{-}LM}$ (a2) as-purchased PEG flakes (a3) as-spun fibers of $PS_{p\text{-}LM}$, $PS_{p\text{-}LM}$-10, and porous $PS_{p\text{-}LM}$-10.

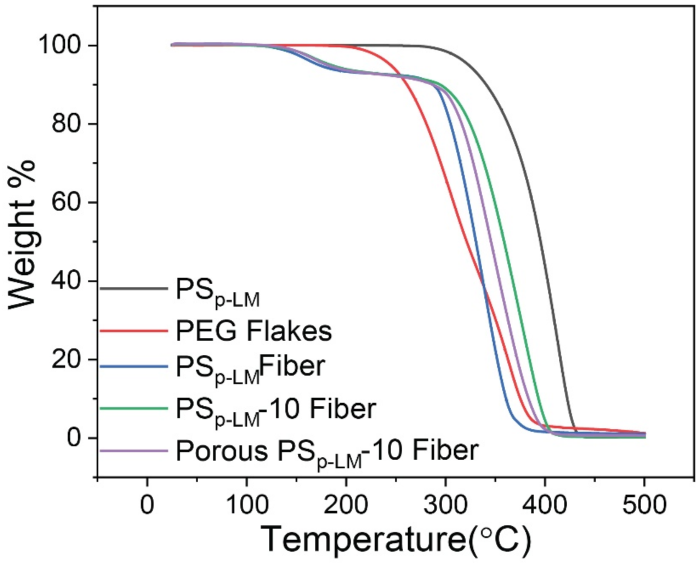

Figure S5. TGA curves of $PS_{p\text{-}LM}$ and PEG flakes as purchased and as-spun fibers of $PS_{p\text{-}LM}$, $PS_{p\text{-}LM}$-10, and porous $PS_{p\text{-}LM}$-10

Table S1. Mechanical properties for obtained and fabricated fibers at varying PEG concentration

	Solvent of DMF	
**Fiber**	**Modulus (MPa)**	**Strength (MPa)**
$PS_{p\text{-}LM}$ - 0	105.278	0.42
$PS_{p\text{-}LM}$ – 1	147.69	0.461
$PS_{p\text{-}LM}$ – 2	159.728	0.5799
$PS_{p\text{-}LM}$ – 3	134.269	0.842
$PS_{p\text{-}LM}$ – 4	159.331	1.091
$PS_{p\text{-}LM}$ – 5	166.123	1.305
$PS_{p\text{-}LM}$ - 10	122.769	1.395
$PS_{p\text{-}HM}$ – 10	110.953	1.563

Tensile test of the shell fiber made of 35 wt% $PS_{p\text{-}LM}$/ DMF/ PEG of (a) 1 wt%, (b) 2 wt%, (c) 3 wt%, (d) 4 wt%, (e)5 wt%, and, (f) 10 wt%, 10mm in length and 1.088 mm in diameter at a constant linear rate of 10 μm. The table above gives the elastic modulus and tensile strength of the fiber.
